# Injection-Molded Parts of Partially Biobased Polyamide 610 and Biobased Halloysite Nanotubes

**DOI:** 10.3390/polym12071503

**Published:** 2020-07-06

**Authors:** David Marset, Celia Dolza, Teodomiro Boronat, Nestor Montanes, Rafael Balart, Lourdes Sanchez-Nacher, Luis Quiles-Carrillo

**Affiliations:** 1 Textile Industry Research Association (AITEX), Plaza Emilio Sala, 1, 03801 Alcoy, Spain; dmarset@aitex.es (D.M.); cdolza@aitex.es (C.D.); 2 Technological Institute of Materials (ITM), Universitat Politècnica de València (UPV), Plaza Ferrándiz y Carbonell 1, 03801 Alcoy, Spain; tboronat@dimm.upv.es (T.B.); nesmonmu@upvnet.upv.es (N.M.); rbalart@mcm.upv.es (R.B.); lsanchez@mcm.upv.es (L.S.-N.)

**Keywords:** PA610, halloysite nanotubes (HNTs), flame retardant, thermomechanical resistance

## Abstract

This works focuses on the development of environmentally friendly composites with a partially biobased polyamide 610 (PA610), containing 63% biobased content, and a natural inorganic filler at the nanoscale, namely, halloysite nanotubes (HNTs). PA610 composites containing 10, 20, and 30 wt% HNTs were obtained by melt extrusion in a twin screw co-rotating extruder. The resulting composites were injection-molded for further characterization. The obtained materials were characterized to obtain reliable data about their mechanical, thermal, and morphological properties. The effect of the HNTs wt% on these properties was evaluated. From a mechanical standpoint, the addition of 30 wt% HNTs gave an increase in tensile modulus of twice the initial value, thus verifying how this type of natural load provides increased stiffness on injection molded parts. The materials prepared with HNTs slightly improved the thermal stability, while a noticeable improvement on thermomechanical resistance over a wide temperature range was observed with increasing HNTs content. The obtained results indicate that high biobased content composites can be obtained with an engineering thermoplastic, i.e., PA610, and a natural inorganic nanotube-shaped filler, i.e., HNTs, with balanced mechanical properties and attractive behavior against high temperature.

## 1. Introduction

Currently, there is great social awareness concerning the use of biopolymers capable of minimizing and/or overcoming the environmental impact of fossil fuels. In the last years consumer habits have changed in a remarkable way, especially to purchase more sustainable products [1,2,3,4]. For this reason, a great interest has been generated in the last few decades to research and use biobased materials that could play a key role in the partial/total replacement of synthetic materials [5,6].

In this context, polyamides have been used since their appearance in the 1930s in a multitude of applications. They were mainly used as fibers, but in recent years, due to their excellent properties, they have found interesting uses in a wide range of technical applications, including engineered injection-molded and extruded parts [7]. Furthermore, in sectors such as the automotive [8] and textile industry [9], polyamide 6 (PA6) has always received great attention thanks to its good mechanical performance and high thermal resistance, which are the result of the strong hydrogen bonds contained in its chemical structure [10]. Polyamides are generally linear and semi-crystalline polymers, with recurrent amide groups as an integral part of the main polymer chain. These are commonly known in the plastic industry as “nylons”, which refer to any polyamide having less than 85% of the amide groups directly connected to two aromatic groups [11].

Thanks to the new trends in the biobased industry, high-performance polymers can be obtained not only from fossil fuels, but can also be partially or fully synthesized from biobased raw materials [12,13]. In particular, the development of biobased polyamides (bio-PA) has gained great interest thanks to the new social awareness in favor of the environment [14]. In this context, castor oil (CO) plays an important role in obtaining bio-PA [15]. This vegetable oil is completely natural, as well as economic. It is a triglyceride with 85–95% ricinoleic acid. CO is available in its triglyceride esters and allows the obtainment of different types of biobased polyamides [16]. In the case of polyamide 6 (PA6), there is a biobased counterpart with different chemical structure. This is polyamide 610 (PA610), with very similar properties, but with the advantage of containing renewable 10 carbon atom-chains (C10) monomers derived from castor oil. In this sense, dicarboxylic acid C10 can easily react with petroleum-based 1,6-hexamethylenediamine (HMDA), obtained from butadiene, by polycondensation to produce aliphatic polyamide 610 (PA610). As a result, PA610 has 60–63% renewable content [17].

On the other hand, the use of fillers and additives is currently a promising approach for improving the range of possible applications of this type of biopolymers [18]. Currently, a large number of these are used such as silicate clays [19], carbon black [20,21], graphite [22,23], graphene [24] or fibers [25,26], with the aim of improving and modifying some properties of polyamides to obtain tailored properties. Moreover, it is necessary to bear in mind that one of the main targets of the polymer industry is to obtain fully renewable and natural products, so that, the search for these products is of key importance [27,28]. Over the past two decades, nanocomposites based on inorganic clay minerals have attracted a great deal of attention. Due to the nanoscale structure, nanocomposites exhibit significant improvements in properties, such as improved mechanical properties, reduced gas permeability, increased thermal stability and improved flame retardancy [29,30,31,32], among others. For this reason, the use of halloysite nanotubes (HNTs) is becoming increasingly important. HNTs are naturally occurring aluminosilicate clays, which have a composition similar to kaolinite. They are naturally produced by the prolonged erosion of alumina silicates and can be found in the form of fine tubular structures with a length of 300–1500 nm, and internal diameter (lumen) and external diameters of 15–100 and 40–120 nm, respectively [33]. With this tubular characteristic, they have a large active surface, and can also be used as containers for various functional reagents such as antioxidants [34]. In recent years, HNTs have become the focus of research as a new type of multifunctional filler to improve mechanical properties [33,35], thermal behavior [35], crystallization [36] and flame retardancy [37,38].

These highly efficient composites could be used to manufacture engineering parts and assemblies with good and balanced mechanical properties, good dimensional stability, and potential fire retardancy properties, since HNTs have been proved to provide this particular feature as above-mentioned. High technological sectors such as the automotive and construction industries are the main candidates for incorporating this type of composites. Currently, there are different direct applications of HNTs as multifunctional additives in polymer matrices. It is worthy to note the increasing use of HNTs in active packaging [39,40], automotive [41,42], construction [43,44], and medicine [33,45] industries, among others.

This study focuses on the use of halloysite nanotubes (HNTs) as filler in partially biobased polyamide 610 (PA610) to analyze how their incorporation affects their mechanical, thermal, and morphological properties. The main aim is to obtain balanced mechanical properties on PA610/HNT composites and evaluate the influence of temperature on both thermal degradation and dynamic-mechanical behavior.

## 2. Materials and Methods

### 2.1. Materials

PA610 was supplied, in the form of pellets (NP BioPA610-201) by NaturePlast (Ifs, France). According to the manufacturer, this is a biobased medium-viscosity injection-grade homopolyamide with a density of 1.06 g/cm^3^ and a viscosity number (VN) of 160 cm^3^/g. This polyamide has 63% renewable content. Halloysite nanotubes were supplied by Sigma Aldrich with CAS number 1332-58-7.

### 2.2. Sample Preparation

PA610 and HNTs were dried at 60 °C for 48 h in a dehumidifying dryer model MDEO from Industrial Marsé (Barcelona, Spain) to remove any residual moisture prior to processing. The two different components were mechanically pre-homogenized in a zipper bag according to compositions in Table 1. The materials were then fed into the main hopper of a co-rotating twin-screw extruder from Construcciones Mecánicas Dupra S.L. (Alicante, Spain). The screws feature a 25 mm diameter, with a length-to-diameter ratio (L/D) of 24. The extrusion process was carried out at 20 rpm. The temperature profile was set (from the hopper to the die) as follows: 215–225–235–245 °C. The different PA610/HNTs composites were extruded through a round die to produce strands and, subsequently, were pelletized using an air-knife unit. In all cases, the residence time was approximately 1 min.

The compounded pellets were, thereafter, shaped into standard samples by injection molding in a Meteor 270/75 from Mateu and Solé (Barcelona, Spain). Two different shapes were injection-molded to obtain samples for further characterization. Dog bone-shaped samples of 150 × 10 × 4 mm^3^ as indicated by ISO 527-1:2012 were obtained for tensile tests. In addition, rectangular 80 × 10 × 4 mm^3^ samples were obtained for other characterizations. The temperature profile in the injection molding unit was 220 (hopper), 225, 230, and 235 °C (injection nozzle). A clamping force of 75 tons was applied while the cavity filling and cooling times were set to 1 and 10 s, respectively. Moreover, a constant filling time of 1 s was maintained for all samples.

### 2.3. Characterization Techniques

#### 2.3.1. Mechanical Tests

Tensile tests were carried out in a universal testing machine ELIB 50 from S.A.E. Ibertest (Madrid, Spain) using injection-molded dog bone-shaped samples. A 5 kN load cell was used and the cross-head speed was set to 5 mm/min. Shore hardness was measured in a 676-D durometer from J. Bot Instruments (Barcelona, Spain). Toughness was also studied on injection-molded rectangular samples by the Charpy impact test with a 1-J pendulum from Metrotec S.A. (San Sebastián, Spain) on notched samples (0.25 mm radius v-notch), following the specifications of ISO 179-1:2010. All tests were performed at room temperature, that is, 25 °C, and at least 6 samples of each material were tested, and their corresponding properties were averaged.

#### 2.3.2. Morphology

The morphology of the fracture surfaces of the PA610/HNTs composite parts, obtained from impact tests, was observed by field emission scanning electron microscopy (FESEM) in a ZEISS ULTRA 55 from Oxford Instruments (Abingdon, UK) microscope. Before placing the samples in the vacuum chamber, samples were sputtered with a gold-palladium alloy in an EMITECH sputter coating SC7620 model from Quorum Technologies, Ltd. (East Sussex, UK). An acceleration voltage of 2 kV was applied.

#### 2.3.3. Thermal Analysis

The main thermal transitions of the injection-molded PA610/HNTs composite parts were obtained by differential scanning calorimetry (DSC) in a Mettler-Toledo 821 calorimeter (Schwerzenbach, Switzerland). An average sample weight ranging from 5 to 7 mg was subjected to a three-stage dynamic thermal cycle as follows: first heating from 20 to 250 °C followed by cooling to 0 °C, and second heating to 350 °C. Heating and cooling rates were set to 10 °C/min. All tests were run in nitrogen atmosphere with a flow of 66 mL/min using standard sealed aluminum crucibles (40 μL). The degree of crystallinity (*χ_c_*) was determined following the equation:

(1) $$\chi_{c}\left(\%\right)=\left[\frac{∆H_{m}}{∆H_{m}^{0}\times \left(1-w\right)}\right]\times 100$$

where ∆*H_m_* (J/g) stands for the melting enthalpy of the sample, ∆*H_m_*_0_ (J/g) represents the theoretical melting enthalpy of a fully crystalline PA610, that is, 197 J/g [46], and *w* corresponds to the weight fraction of HNTs in the formulation.

Thermogravimetric analysis (TGA) was performed in a LINSEIS TGA 1000 (Selb, Germany). Samples with an average weight between 5 and 7.5 mg were placed in standard alumina crucibles of 70 µL and subjected to a heating program from 30 to 700 °C at a heating rate of 10 °C/min in air atmosphere. The first derivative thermogravimetric (DTG) curves were also determined, expressing the weight loss rate as function of time. All tests were carried out in triplicate to obtain reliable results.

#### 2.3.4. Thermomechanical Characterization

Dynamical mechanical thermal analysis (DMTA) was carried out in a DMA-1 dynamic analyzer from Mettler-Toledo (Schwerzenbach, Switzerland), working in single cantilever flexural conditions. Injection-molded samples with dimensions of 20 × 6 × 2.7 mm^3^ were subjected to a dynamic temperature sweep from −60 to 160 °C at a constant heating rate of 2 °C/min. The selected frequency was 1 Hz and the maximum flexural deformation or deflection was set to 10 µm.

The dimensional stability of the PA610/HNTs composites was studied by thermomechanical analysis (TMA) in a Q400 thermomechanical analyzer from TA Instruments (New Castle, DE, USA). The applied force was set to 0.02 N and the temperature program was scheduled from −60 to 160 °C in an air atmosphere (50 mL/min) at a constant heating rate of 2 °C/min. The coefficient of linear thermal expansion (CLTE) of PA610/HNTs composites, both below and above *T*_g_, were determined from the change in dimensions versus temperature.

## 3. Results

### 3.1. Mechanical Properties of PA610/HNTs Composite Parts

The mechanical properties obtained from mechanical tests are shown in Table 2. In particular, the values of tensile modulus (E), maximum tensile stress (σ_max_) and elongation at break (ε_b_) of PA610 composites with different wt% of HNTs obtained from tensile tests are reported. In relation to neat material, PA610 shows E and σ_max_ values of 1992 MPa and 48.1 MPa respectively, while ε_b_ is 250%. These values indicate PA610 has moderate-to-high stiffness and strength, while its deformation is very high, being materials very suitable for technical applications due to a high toughness. On the other hand, the incorporation of HNTs induced a significant increase in the rigidity of the injection-molded samples. In particular, the E increased from 1992 MPa to 2716 MPa, for the addition of 10 wt% HNTS, to 3230 MPa for 20 wt% load, and to 4431 MPa for 30 wt% HNTs. In the case of 30% HNTs this means an increase of more than double the initial stiffness. It seems that with low percentages, polyamide 610 behaves similarly to non-biobased PA6. Other authors such as Handge et al. [47] showed that for petroleum-derived polyamides such as PA6, the incorporation of high HNTs loadings led to a great increase in the stiffness. They reported a tensile modulus value of 4000 MPa for HNTs loading close to 30 wt%. These results confirm how the stiffness of polyamide 610 is very similar to the conventional polyamide 6, with very similar results after the incorporation of HNTs.

On the other hand, addition of HNTs into the PA610 matrix produces a slight decrease in the value of σ_max_. Interestingly, the lowest value is 38.5 MPa with the addition of 10 wt% HNTs, compared to 48 MPa for neat material. However, addition of 30 wt% HNTs to PA610 gives a tensile strength of 43 MPa, which is slightly lower than that of neat PA610. This initial reduction may be due to the PA610 structure, since other authors have reported improvements in the maximum tensile strength of PA6 filled with HNTs [47]. However, Francisco et al. [48] reported similar results as seen in this work with biobased polyamide 11. They show how, depending on the composition of the polyamide, the tensile strength (maximum stress) can be affected by different HNTs loadings. Finally, it can be appreciated how addition of HNTs promote a dramatic decrease in the elongation at break, from 250% (neat PA610) to values between 11–13% for all PA610-HNTs compositions. This behavior is typical of particle-filled polymers. It is worthy to note that the tensile modulus depends on both the applied stress and the corresponding elongation in the linear region, since it stands for the stress to strain ratio in this region (E = σ/ε). Therefore, as tensile tests have revealed, the decrease in the tensile strength is not as pronounced as that obtained for the elongation at break. Since the elongation (ε) is placed in the denominator, it is expectable an increase in the E value, as described previously. Boonkongkaew et al. [38] showed similar results by incorporating HNTs to thermoplastic PA6, leading to a reinforcement effect on the obtained composites. In addition, the improvement on tensile modulus and strength caused a dramatic drop in elongation at break. This effect is not as pronounced if the filler shows somewhat compatibility. HNTs are highly hydrophilic while PA610, despite it shows some moisture sensitiveness, offers a hydrophobic behavior. This difference does not allow strong polymer–particle interactions and therefore, the filler promotes some stress concentration phenomenon.

Following with the mechanical properties, the Shore D hardness values follows a similar trend, offering higher hardness as the HNTs content increases. In this sense, it changes from 77.4 for neat PA610 to 80.2 for composites with 30 wt% HNTs. This increase is directly related to the improvement in stiffness provided by the addition of an inorganic filler into PA610 matrix. The incorporation of inorganic nano-fillers as fire retardants directly increases the hardness of the obtained composites. TF da Silva et al. [49], showed that there is generally a tendency to increase the hardness of nanocomposites by increasing the content of fire-retardant fillers such as ATP or HNTs. In terms of impact strength, composites with HNTs offer decreased values. It can be observed how the value of 6.5 kJ/m^2^ (neat PA610) changes to 1.8 and 1.5 kJ/m^2^, for 10 wt% of HNTs and 20 and 30 wt% respectively. This reduction is due to the possible stress concentration phenomena that are generated in the matrix due to the dispersed HNTs as above-mentioned. This parameter could be relevant when selecting this type of composites in applications that require high toughness. Despite this, it is worthy to note, that it was necessary to use notched samples to break them and obtain the impact strength.

### 3.2. Morphology of PA610/HNTs Composites

**Figure 1:**
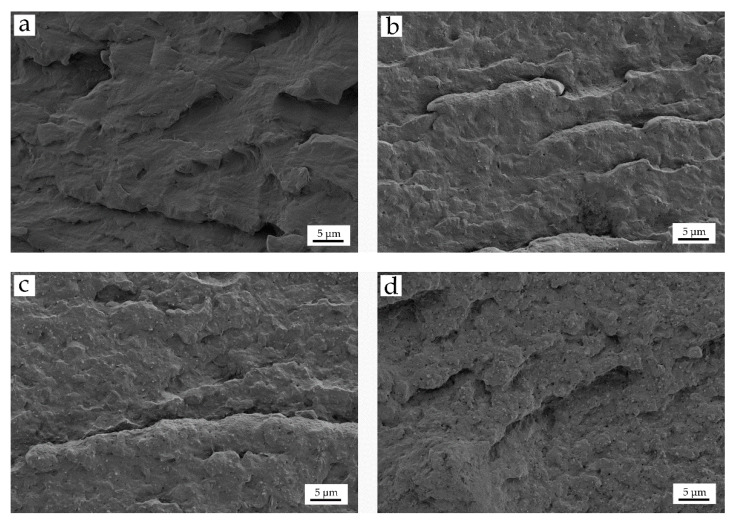
Field emission scanning electron microscopy (FESEM) images at 2000× of the fracture surfaces of PA610/HNT composites: (**a**) PA610; (**b**) PA610/10HNTS; (**c**) PA610/20HNTS and (**d**) PA610/30HNTs.

Figure 1 depicts the FESEM images corresponding to the impact fracture surfaces of the specimens, in order to analyze how the incorporation of the nano-loads affects their structure. Figure 1a shows the morphology of the neat PA610 fracture surface, at 2000×. In this image, a relatively rough morphology can be clearly seen. The small steps that give rise to this roughness do not have sharp edges or straight crack fronts. This morphology corresponds to the typical fracture of ductile polymers. This fracture morphology corroborates the previous mechanical characterization results of PA610, with high elongation at break which is representative for its high plastic deformation. Polyamides usually have this type of rough and cavernous surface structures after fracture, verifying the excellent technical properties of the material [50,51].

**Figure 2:**
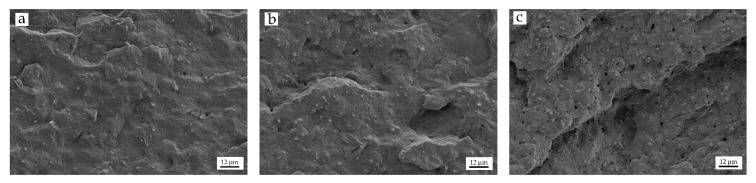
Field emission scanning electron microscopy (FESEM) images at 5000× of the fracture surfaces of PA610/HNT composites: (**a**) PA610/10HNTs; (**b**) PA610/20HNTs and (**c**) PA610/30HNTs.

On the other hand, the images corresponding to PA610 with different HNTs content, show a fracture surface characterized by a structure with a homogeneous polymeric matrix phase and a dispersed HNTs particles phase. The dispersed phase has a fine distribution, with higher density than the higher wt% HNTs in the composite. During the fracture process, part of this dispersed phase is pulled out of the matrix, giving rise to the formation of micro-holes visible on the corresponding images. In addition, the finely dispersed particles interrupt the matrix continuity. This is responsible for a poor stress transmission when the material is subjected to external stresses. This has a negative effect on plastic deformation. Both the dispersed particles and the micro-holes cause a marked loss of ductility of the PA610/HNTs composites, as seen in the previous section. Figure 1b,c shows the FESEM images corresponding to the fracture surfaces of composites containing 10 and 20 wt% of HNTs, respectively. In both cases, a good filler dispersion can be observed, however a smoother surface with less roughness can be seen, which in in accordance to the above-mentioned reduction of the ductile properties on PA610/HNT composites. In this context, Boonkongkaew et al. [52], showed how the incorporation of low HNTs loading into a polyamide 6 matrix revealed a homogeneous dispersion of individual isolated nanotubes with an average diameter of ~60 nm in PA6 composites containing 5 to 10 wt% HNTs. This phenomenon could be observed in more detail in Figure 2a, where at low HNTs loading, a uniform dispersion with a low amount of agglomerates can be seen. In Figure 2b, it can be seen how for 20 wt% HNTs, there is still a good dispersion, but with some specific agglomerates due to the large amount of load incorporated.

Figure 2c shows (in more detail than Figure 1d) a larger number of nanotubes, with a good dispersion, but with a higher density of aggregates and micro-holes. With high wt% HNTs, the generation of this type of aggregate is common, as it has been observed in other studies. Sahnoune et al. [53], reported how during the incorporation of HNTs into PA11, a homogeneous and uniform dispersion appears for HNTs, most of them dispersed at the nanometer scale. However, some particles were visible as aggregates, but with very small sizes. Furthermore, they also showed how a certain number of holes were generated.

In this work, PA610/HNTs composites with different HNTs content, show an excellent dispersion of the nanotubes. This phenomenon is due to the fact that HNTs have a unique surface chemical property due to the multi-layer structure, with only a few hydroxyl groups located on the external surface of the nanotubes [54]. Therefore, HNTs can be easily dispersed in a non-polar polymer matrix, due to the weak secondary interactions between the hydrogen-bonded nanotubes and the Van der Waal forces [55]. In addition, the high polarity of the polyamide (compared with other polymers) matrix allows efficient dispersion of the nanotubes by forming H-bonds between the polymer and the filler. Nevertheless, these interactions are not strong and do not allow good stress transfer which has negative effect on cohesion-related properties such as tensile strength, and specifically on elongation at break.

### 3.3. Thermal Properties of PA610/HNTs Composites

**Figure 3:**
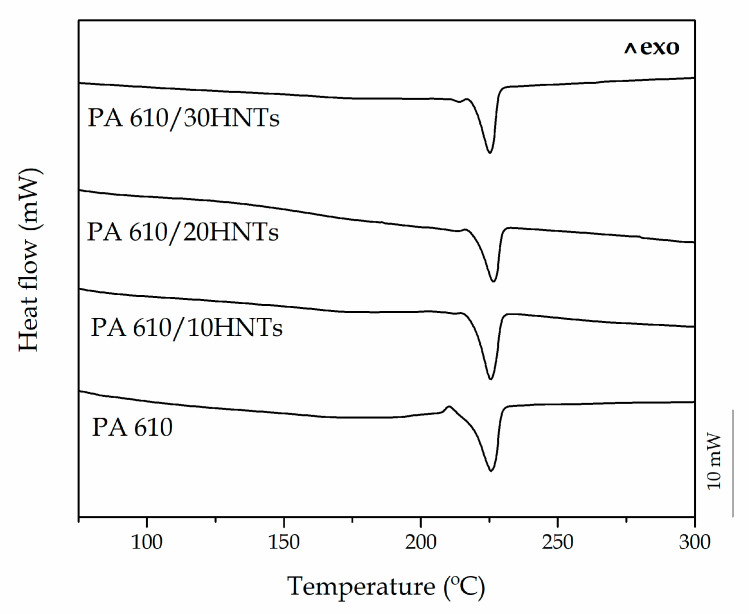
Differential scanning calorimetry (DSC) thermograms of PA610 composites with different HNTs loading.

Figure 3 shows the DSC thermograms obtained during the second heating cycle of PA610 composites with different HNT loads. In addition, Table 3 summarizes the main data obtained during the thermal characterization.

The PA610 thermogram showed a single endothermic peak at 224 °C, corresponding to the polymer melting process. The melting enthalpy, 62.9 J/g, allows the calculation of the degree of crystallinity (*χ_c_*) of PA610, with a value of 32%. Similar results were reported by Wu et al. [56], who showed a melting peak around 223 °C and a *χ_c_* of 34%. However, both neat PA610 and the composite with 30 wt% HNTs show a slight peak before melting, which may be linked to the presence of different crystalline forms where different crystalline lamellae coexist, i.e., α, β and γ. These types of peaks are much more noticeable in non-renewable resource polyamides 6 [57], however, the PA610 with the addition of nano-fillers, appears to have a slight double spike in melting. In this sense, Pai et al. [58] showed similar results in a PA610, with a small double peak, which was attributed to the formation of two different morphological species related to different forms of crystalline lamellae. This type of behavior is due to the reorganization of the lamellae and is related to their thickness. The smaller melting peak is associated with the thin films formed during cooling, while the main melting temperature is due to the melting of the thicker films during the heating process [59].

The incorporation of HNTs to the PA610 has no significant effect on the thermal behavior of the developed composites. The characteristic DSC curves of PA610/HNTs are very similar to those obtained for neat PA610. The small variation of *T*_m_, in only 1 °C to that of neat PA610, indicates that the addition of different wt% HNTs to PA610 does not influence its thermal transitions. With respect to the obtained crystallinity, the incorporation of HNTs means a slight decrease in the crystallinity of PA610. With 10 wt% HNTs the *χ_c_* decreases to 24.5% with respect to 31.9% of neat PA610. On the other hand, the increase in the HNTs content in composites, practically does not influence the crystallinity with an almost constant value of 25%. This means that the addition of this type of filler does not favor the formation of ordered regions, or nucleant effect. In particular, Jeong et al. [59] showed that P-MWNT (pristine multiwalled carbon nanotubes) slightly reduce the crystallinity of composites with PA610, which means in some cases a worse distribution of the load, however, in the studied case and as seen above, the HNTs manage to disperse well in the biopolyamides.

In this sense, Zhang et al. [60], showed that the incorporation of montmorillonite (MMT) can modify the crystallization of biopolyamides. In addition, the physical barrier of the silicate layers affected the second melting peak more than the first one. Therefore, it can be noted that the incorporation of high amounts of HNTs nanotubes could modify the folding process in the biopolymer, thus generating the segmental immobilization of polymer chains, and directly creating a double melting peak.

**Figure 4:**
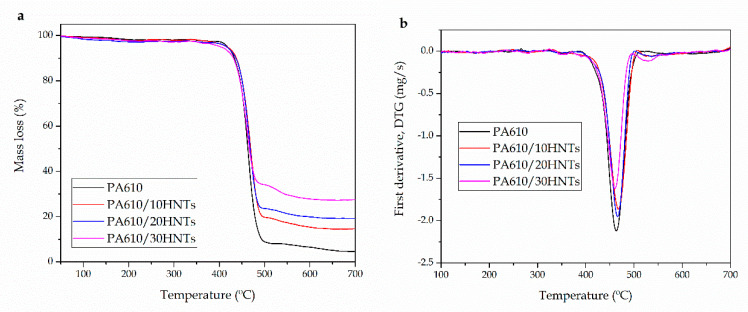
(**a**) Thermogravimetric analysis (TGA) curves and (**b**) first derivative (DTG) of polyamide 610 (PA610) composites with different HNTs loadings.

The influence of HNTs on the overall thermal stability of PA610 was investigated by thermogravimetry (TGA). The obtained TGA thermograms are shown in Figure 4, while the most relevant properties are summarized in Table 4. It should be noted that *T*_5%_ corresponds to the characteristic temperature for a 5 wt% mass loss and is representative for the start of thermal degradation. *T*_5%_ for neat PA610 was 417 °C, while the temperature corresponding to the maximum degradation rate (*T*_deg_), obtained from the peak maximum un DTG plots was 461 °C. Moran et al. [7] reported similar degradation parameters for PA410 and PA610, where the degradation temperature was above 440 °C. These results are very interesting since they indicate the PA degradation occurs at high temperatures compared to commodities and some engineering plastics. In relation to the degradation of this type of biopolyamide, Quiles-Carrillo et al. [61], showed how in general bio-PA are thermally stable up to 320–340 °C, showing similar thermal degradation profiles [62,63]. It was particularly noted that this mainly involves a transfer reaction mechanism from β-CH, which produces ketoamides as the main degradation products [64]. Furthermore, in general, a high moisture content in the samples was not observed as there was no significant weight loss below 100 °C.

Regarding PA610/HNTs composites, presence of HNTs provide a slight increase in both *T*_5%_ and *T*_deg_ of 0.2–2 °C and 1–5 °C, respectively. In particular, the composite containing 10 wt% HNTs had the highest thermal stability, showing a *T*_deg_ value of 466.6 °C. On the other hand, composites with 30 wt% HNTs did not show improved thermal stability but remained constant. The improvement in thermal stability related to the addition of 10 and 20 wt% HNTs, could be related to the generation of a mass transport barrier exerted by the inorganic filler to the volatiles produced during the polymer decomposition [61]. In this context, the incorporation HNTs to PA610 offer similar results to other studies. Shen et al. [65], showed how the addition of MWCNT improved the thermal stability of PA6 by a free radical scavenging effect of the fillers.

On the other hand, degradation behavior by TGA analysis has been generally used to check the residual amount of HNTs added to a polymer matrix. It can be seen how the residual mass of the compositions are almost coincident to the nominal HNTs content. HNTs are organic compounds and offer improver thermal degradation properties compared to polymers.

### 3.4. Thermomechanical Properties of PA610/HNTs Composites

**Figure 5:**
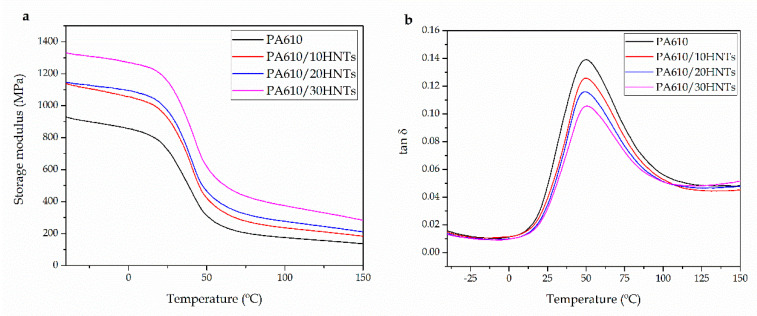
(**a**) Storage modulus (E’) and (**b**) dynamic damping factor (tan δ) of polyamide 610 (PA610) composites with different HNTs loading.

Figure 5 shows the thermomechanical evolution of PA610 and how it is affected by the incorporation of HNTs. Figure 5a shows the evolution of the storage modulus (E′) as the temperature increases. Neat PA610 shows an E’ value of 860 MPa below its *T*_g_, while at 100 °C (above its *T*_g_) it decreases to a value of 175 MPa. Very similar values have been reported by other authors, for this type of biopolyamides [66].

Below the *T*_g_, the addition of 10 wt% HNTs produces an increase of 200 MPa on E’, thus corroborating the increase in stiffness, previously reported by tensile tests. On the other hand, the addition of 20 wt% of HNTs seems to maintain the stiffness values very close to those of composites with 10 wt%, while the incorporation of 30 wt% generates a clear increase in E’ of more than 400 MPa, compared to neat PA610. In this context, addition of HNTs positively contributed to increase in the material’s stiffness, both below and above the characteristic *T*_g_ of the base material. This phenomenon is closely related to the good dispersion achieved with this type of load, which greatly favors the increase in rigidity related to inhibition of plastic deformation as observed by tensile tests. Marques et al. [67] showed how the incorporation of HNTs provided a significant increase in the rigidity of polypropylene (PP) matrix. They demonstrated that the presence of HNTs in the polymer matrix had a reinforcing effect, possibly due to the good filler dispersion into the matrix. These results are very similar to those obtained in this work

Additionally, in Table 5 and Figure 5b, the *T*_g_ values of each PA610/HNTs composite together with the storage modulus (E’) values at different temperatures are shown. If the *T*_g_ of the obtained materials is analyzed, it can be seen how the addition of HNTs (in particular 30 wt% loading) in the mixture means a slight increase in the base *T*_g_ of PA610, changing from 49.6 °C to 50.6 °C. This increase may not be significant; however, this alteration may be interesting when it comes to commenting an improvement in the interaction between the polymer chains and the load. In this context, some works have reported how the addition of HNTs in a polyamide matrix can have two opposite effects on the glass transition temperature of the base polymer. On one hand, a decrease in *T*_g_ associated with reduced interactions between PA chains can be seen due to the presence of the fillers. This phenomenon improves the mobility of the polymer chains [55,68,69]. On the other hand, an increase in *T*_g_ may appear. This could be caused by a restriction of the segmental movement of the PA chains located near the surface of the nanotubes. This can only be possible if the hydrogen bonding interactions between the amine groups and the hydroxyl groups located at the surface of HNTs are strong enough. Lecouvet et al. [36] showed how in the case of PA12, well-dispersed HNTs reduce the intermolecular hydrogen bond between PA chains and dominate the *T*_g_ reduction effect. However, above 10 wt% HNTs loading, this effect is compensated by the interactions between HNTs and PA12 chains, resulting in an overall increase in *T*_g_.

## 4. Conclusions

This work shows that the incorporation of nano-loads with potential fire retardancy capabilities, such as HNTs, can be effectively used as novel reinforcement in partially biobased PA610 manufactured by extrusion and subsequent injection molding.

PA610/HNTs composites containing 30 wt% HNTs showed a twofold increase in the tensile modulus, while the tensile strength was not reduced in a remarkable way. This increase is in stiffness is also related to a dramatic decrease in elongation at break which changes from 250% (neat PA610) to values of 11–12% for all PA610/HNT composites. Regarding the impact strength, it is worthy to note an important decrease by the addition of HNTs. This reduction could be due to the potential stress concentration that are generated in the matrix due presence of finely dispersed hydrophilic HNTs into the hydrophobic PA610 matrix.

On the other hand, the morphology shows an internal structure with a homogeneous polymeric matrix phase and a dispersed phase of HNTs. In the samples analyzed with different wt% HNTs, an excellent dispersion of the nanotubes was observed. This phenomenon is due to the fact that HNTs have a unique surface chemical property due to the multi-layer structure with hydroxyl groups located on the surface of the nanotubes. The thermal analysis revealed how the incorporation of HNTs did not have a significant impact on the characteristic thermal properties of PA610. Despite this, a slight improvement of the thermal stability of 5 °C was observed. The improvement could be related to the generation of a mass transport barrier (tortuous path) to the generated volatiles, produced by the inorganic filler.

Finally, the incorporation of HNTs implies a clear improvement in the stiffness. Addition of 30 wt% HNTs increases the storage modulus (E’) by 400 MPa. The incorporation of HNTs means a great increase in the material’s stiffness, both below and above the *T*_g_ of the partially biobased PA610 matrix. This phenomenon is closely related to a relatively good filler dispersion obtained by a first extrusion/compounding process and a second injection molding process. The use of PA610 and HNTs can contribute positively to the development of sustainable polymer technologies by decoupling raw materials from fossil fuels. As HNTs have remarkable fire retardancy properties, the mechanical, thermal and thermomechanical properties obtained in this work could serve as the base for the development of environmentally friendly plastic fire retardant formulations with balanced mechanical and thermal properties for uses in technological applications.

## Figures and Tables

**Table 1:** Summary of compositions according to the weight percentage content (wt%) of polyamide 610 (PA610) and halloysite nanotubes (HNTs).

Code	PA610 (wt%)	HNTs (wt%)
PA610	100	0
PA610/10HNTs	90	10
PA610/20HNTs	80	20
PA610/30HNTs	70	30

**Table 2:** Summary of mechanical properties of the injection-molded parts of polyamide610 (PA610) with HNTs, in terms of: tensile modulus (E), maximum tensile strength (σ_max_), and elongation at break (ε_b_), Shore D hardness, and impact strength.

Parts	E (MPa)	σ_max_ (MPa)	ε_b_ (%)	Shore D Hardness	Impact Strength (kJ/m^2^)
PA610	1992 ± 80	48.1 ± 2.1	250.0 ± 55.2	77.4 ± 0.6	6.5 ± 0.3
PA610/10HNTs	2716 ± 120	38.5 ± 2.4	11.4 ± 0.9	78.2 ± 0.5	1.8 ± 0.2
PA610/20HNTs	3230 ± 140	42.8 ± 1.4	12.9 ± 1.0	79.4 ± 0.5	1.5 ± 0.3
PA610/30HNTs	4431 ± 155	43.1 ± 2.3	11.3 ± 0.9	80.2 ± 0.8	1.5 ± 0.2

**Table 3:** Main thermal parameters of the injection-molded parts of polyamide 610 (PA610) with HNTs in terms of: melting temperature (*T*_m_), normalized melting enthalpy (∆*H_m_*), and percentage of crystallinity (*χ_c_*).

Parts	*T*_m_ (°C)	∆*H_m_* (J/g)	*χ_c_* (%)
PA610	224.2 ± 1.5	62.9 ± 0.9	31.9 ± 0.8
PA610/10HNTs	225.4 ± 1.1	43.4 ± 1.1	24.5 ± 0.9
PA610/20HNTs	225.6 ± 0.9	40.3 ± 1.2	25.6 ± 1.0
PA610/30HNTs	225.8 ± 0.8	34.6 ± 0.9	25.1 ± 0.8

**Table 4:** Main thermal degradation parameters of polyamide 610 (PA610) composites with different HNTs loadings of in terms of: temperature at a mass loss of 5 wt% (*T*_5%_), maximum degradation rate temperature (*T*_deg_), and residual mass at 700 °C.

Parts	*T*_5%_ (°C)	*T*_deg_ (°C)	Residual Weight (%)
PA610	417.4 ± 1.5	461.5 ± 1.8	2.9 ± 0.5
PA610/10HNTs	418.9 ± 1.2	466.6 ± 1.7	12.9 ± 0.9
PA610/20HNTs	419.6 ± 1.1	465.7 ± 0.9	20.8 ± 1.6
PA610/30HNTs	417.6 ± 0.9	461.8 ± 1.2	28.9 ± 1.2

**Table 5:** Main thermomechanical parameters of the polyamide 610 (PA610) composites with different HNTs loadings.

Parts	E’ (MPa) at 0 °C	E’ (MPa) at 100 °C	*T*_g_ (°C)
PA610	860 ± 18	175 ± 5	49.6 ± 0.8
PA610/10HNTs	1060 ± 17	230 ± 8	49.7 ± 0.9
PA610/20HNTs	1100 ± 25	275 ± 7	49.6 ± 1.1
PA610/30HNTs	1270 ± 22	380 ± 14	50.6 ± 1.0
